# Membrane properties and coupling of macroglia in the optic nerve

**DOI:** 10.1016/j.crneur.2024.100137

**Published:** 2024-08-14

**Authors:** Nine Kompier, Marcus Semtner, Sophie Walter, Natali Kakabadze, Christian Steinhäuser, Christiane Nolte, Helmut Kettenmann

**Affiliations:** a Max-Delbrück-Center for Molecular Medicine in the Helmholtz Association, Dep. of Cellular Neurosciences, 13125, Berlin, Germany; b Institute of Cellular Neurosciences, Medical Faculty, University of Bonn, Venusberg-Campus 1, 53127, Bonn, Germany; c Shenzhen Institutes of Advanced Technology, Chinese Academy of Sciences, Shenzhen, China; d Charité Universitätsmedizin, Experimental Ophtalmology, Campus Virchow, Augustenburger Platz 1, 13353, Berlin, Germany; e Department of Pathology, NYU Langone Medical Center, 550 First Avenue, NY, 10016, New York, USA; f Free University of Berlin, Institute for Biology, Virchowweg 6, 10117 Berlin

**Keywords:** Mouse optic nerve, Astrocyte, NG2 glia, In-situ patch clamp, Cell coupling, Transgenic reporter mouse

## Abstract

We established a longitudinal acute slice preparation of transgenic mouse optic nerve to characterize membrane properties and coupling of glial cells by patch-clamp and dye-filling, complemented by immunohistochemistry. Unlike in cortex or hippocampus, the majority of EGFP + cells in optic nerve of the hGFAP-EGFP transgenic mouse, a tool to identify astrocytes, were characterized by time and voltage dependent K^+^-currents including A-type K^+^-currents, properties previously described for NG2 glia. Indeed, the majority of transgene expressing cells in optic nerve were immunopositive for NG2 proteoglycan, whereas only a minority show GFAP immunoreactivity. Similar physiological properties were seen in YFP + cells from NG2-YFP transgenic mice, indicating that in optic nerve the transgene of hGFAP-EGFP animals is expressed by NG2 glia instead of astrocytes. Using Cx43kiECFP transgenic mice as another astrocyte-indicator revealed that astrocytes had passive membrane currents. Dye-filling showed that hGFAP-EGFP+ cells in optic nerve were coupled to none or few neighboring cells while hGFAP-EGFP+ cells in the cortex form large networks. Similarly, dye-filling of NG2-YFP+ and Cx43-CFP+ cells in optic nerve revealed small networks. Our work shows that identification of astrocytes in optic nerve requires distinct approaches, that the cells express membrane current patterns distinct from cortex and that they form small networks.

## Highlights

• Astrocytes from optic nerve and cortex display distinct electrophysiological properties.
• NG2 cells from optic nerve and cortex have similar electrophysiological properties.
• Both astrocytes and NG2 cells in the optic nerve are coupled by gap junctions, but to a limited extent.
• In the optic nerve, the hGFAP-EGFP transgene is predominantly targeted to NG2 cells.

## Graphical abstract

**Figure:**
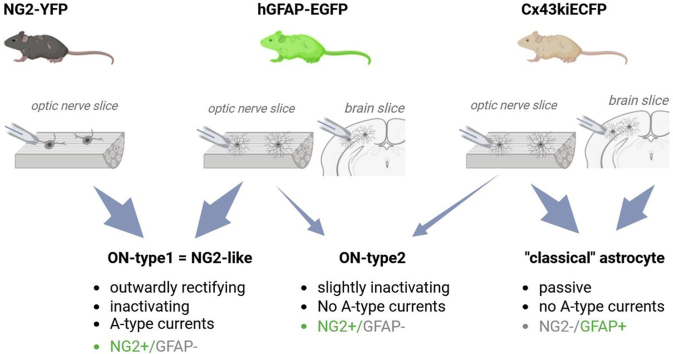

## 1 Introduction

Astrocytes and oligodendrocytes, the macroglial cells of the central nervous system, are described as electrically passive and non-excitable cells (Kettenmann et al., 2004). Astro- and oligodendroglia have integral roles in many aspects of CNS formation and a wide range of physiological functions in normal and pathological conditions, including myelin formation, structural and metabolic support of neurons, maintenance of extracellular ionic homeostasis, and neurotransmitter removal (Allen et al., 2018; McNeill et al., 2021; Simons et al., 2015). More recently, an additional class of glial cells, characterized by the expression of the membrane chondroitin sulfate proteoglycan NG2, was recognized (Nishiyama, 2001; Butt et al., 2002, 2005; Dawson et al., 2003). In white matter, most of the NG2 glia are precursor cells that give rise to oligodendrocytes, but in grey matter the majority retains its NG2 phenotype throughout adulthood (Dimou et al., 2008; Trotter et al., 2010; Kang et al., 2010).

Astrocytes lack voltage gated channels and are characterized by passive membrane properties with high resting membrane potential and low input resistance (Steinhäuser et al., 1992; Matyash et al., 2010; Bedner et al., 2020). In contrast, NG2 glia express voltage gated K^+^ channels including those of the A-type (Bedner et al., 2020).

Astrocytes are coupled amongst themselves, and as panglial networks to oligodendrocytes through gap-junction channels composed of connexin (Cx) proteins. Astrocytes and oligodendrocytes are characterized by expression of distinct connexin isoforms (Griemsmann et al., 2015; Giaume et al., 2010; Kunzelmann et al., 1999; Nagy et al., 2000). There is only limited information whether NG2 cells are incorporated in these panglial networks (Griemsmann et al., 2015; Maglione et al., 2010). Depending on the CNS region, the coupled networks vary in size, shape and composition. Networks in grey matter are mainly composed of astrocytes (e.g. in neocortex, hippocampus) or astrocytes and oligodendrocytes (called panglial coupling, e.g. in thalamus) and often exceed >100 cells (Griemsmann et al., 2015; Claus et al., 2018). In white matter, such as corpus callosum, networks are also formed by oligodendrocytes and astrocytes but these networks are typically smaller than in grey matter (Maglione et al., 2010; Meyer et al., 2018). In spinal cord, coupling between oligodendrocytes was observed in grey matter, but not in white matter (Pastor et al., 1998).

Rodent optic nerve as a typical CNS white matter tract has been intensely used as model tissue to study energy metabolism and axonal-glial interactions (Brown et al., 2019; Butt et al., 2004) for a review). Glial cell coupling has been characterized by intracellular injection of dyes through sharp electrodes (Butt et al., 1989, 1993). These studies revealed occasional dye-coupling both between presumable oligodendrocytes and astrocytes, however, coupled cells were identified based on their morphological features but not confirmed by cell-type specific markers.

Here, we employed different glial cell type-specific reporter mouse lines and developed an acute slice preparation of adult mouse optic nerve that for the first time allows recording membrane currents and dye-filling employing the patch-clamp technique to characterize glial cell electrophysiological properties and coupling in this white matter tract. We found that cells express membrane current patterns distinct from cortex or hippocampus and form only small panglial coupled networks. Interestingly, in the hGFAP-EGFP transgenic mouse line, EGFP is not targeted to astrocytes, but rather to NG2 glia, a phenomenon that has also previously been observed in hippocampus (Matthias et al., 2003).

## 2 Results

### 2.1 GFAP-EGFP+ cells in the optic nerve comprise two populations electrophysiologically distinct from typical astrocytes in forebrain

The transgenic mouse line expressing EGFP under control of the hGFAP promoter is well established to identify astrocytes in hippocampus and cortex (Matthias et al., 2003; Hirrlinger et al., 2005; Nolte et al., 2001). We set up an acute slice preparation of longitudinally sliced optic nerves (ON; see Materials and Methods) and studied hGFAP-EGFP+ cells by the patch clamp technique (N = 43 mice, n = 68 cells; Fig. 1A). Cells were clamped at a series of de- and hyperpolarizing voltages ranging from −170 mV to +60 mV from a holding potential of −70 mV. Interestingly, individual current profiles and the current-voltage relationships (IV) of hGFAP-EGFP+ cells in the ON were not homogeneous, and analysis revealed two different patterns of membrane currents (Fig. 1B and C). The major population (n = 51, 75%), hereinafter referred to as ON type 1, was outwardly rectifying with a median outward conductance of G_out_ = 10.2 (6.9/14.2) nS, and a statistically different median inward conductance of G_in_ = 3.9 (1.3/7.3) nS (*p* < 0.0001). Currents reversed at −66.3 (−77.2/-59.1) mV and displayed a median membrane resistance of 115.3 (86.3/172.6) MΩ. Outward currents showed voltage-dependent inactivation at potentials more positive than −60 mV, and inward currents declined negative to −120 mV due to channel plug by intracellular Na^+^ (Schröder et al., 2002) (Fig. 1B). These properties clearly differ from GFAP-EGFP+ cells in the cortex (GFAP_Cx; N = 7 mice, n = 17 cells) that were characterized by linear IV relationships with much higher conductances (G_in_ = 73.8 (36.6/81.2) nS; G_out_ = 64.1 (44.7/76.7) nS; Fig. 1B and C), more negative reversal potentials (−79.8 (−81.4/-76.4) mV; *p* = 0.0025 vs. ON type 1) and significantly smaller membrane resistances (14.1 (12.5/25.3) MΩ; *p* < 0.0001 vs. ON type 1; Fig. 1D).

**Fig. 1:**
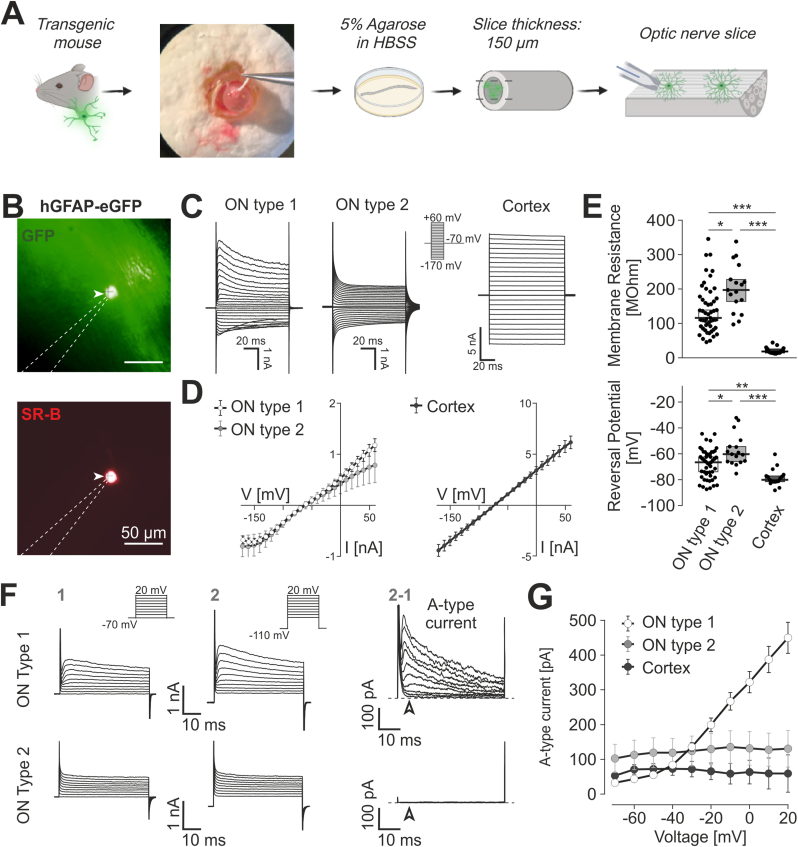
GFAP-EGFP + cells in the optic nerve comprise two populations electrophysiologically distinct from typical astrocytes in forebrain **(A)** Schematic representation of optic nerve slicing procedure. The eye is removed from the transgenic mouse. The second image shows an eye with the optic nerve still attached (held by the forceps). Slicing procedure is detailed in methods. **(B)** Fluorescent images of a patched-clamped cell (arrowhead) in an optic nerve longitudinal slice of a hGFAP-EGFP mouse. *Top:* EGFP fluorescence image. *Bottom:* same cell labelled with sulforhodamine B after 20 min dialysis via the patch-pipette. Scale Bar: 50 μm. Dotted line: patch pipette. **(C)** Representative membrane currents of GFAP-EGFP+ cells in response to voltage pulses (*inset*) ranging from −160 mV to +60 mV in optic nerve (ON type 1 and type 2 cells) and cortex. **(D)** Average current-voltage relationships of optic nerve type 1 (n = 51, left), type 2 cells (n = 17, left) and cortical astrocytes (n = 17; right) (See also Figure S1). **(E)** Box plots (median with 25/75% CI) of membrane resistances (upper panel) and reversal potentials (lower panel) for GFAP-EGFP+ cells in optic nerve (ON type 1, ON type 2) and cortex (See also Figure S1). **(F)** Detection of A-type currents. Currents resulting from a voltage protocol as indicated by insets were recorded from optic nerve GFAP-EGFP+ cells (ON type 1 and ON type 2) from a holding potential of −70 mV (1) and a holding potential of −110 mV (2). The fast-decaying A-type currents resulting from subtraction (2–1) of the currents collected from −70 mV from those collected from −110 mV can be detected in ON type 1 cells (upper row) but not in ON type 2 cells (lower row) (See also Figure S2). **(G)** Average current density of A-type currents in ON type 1 (white, n = 35), ON type 2 (light grey, n = 13) and cortex (black, n = 15) plotted as a function of voltage.

The second population of GFAP-EGFP+ cells in the optic nerve (ON type 2; n = 17, 25%) displayed, like cortical astrocytes, rather linear IV relationships (G_in_ = 5.4 (3.3/5.9) nS; G_out_ = 3.2 (2.4/5.3) nS; Fig. 1B and C). However, membrane resistances (183.8 MΩ (121.9/195.7)) were significantly different from cortical GFAP + cells (*p* < 0.0001), and from optic nerve type 1 cells (*p* = 0.03). The reversal potentials of ON type 2 cells (60.1 (−66.0/51.7) mV) were more positive as compared to ON type 1 cells (*p* = 0.0275) and to cortical GFAP + cells (*p* < 0.0001) (Fig. 1D). Membrane properties of hGFAP-EGFP+ cells in the hippocampus (N = 7 mice, n = 18 cells; Suppl. Fig. 1) were expectedly similarly passive as in the cortex, and thus, different from type 1 and type 2 cells in the optic nerve. We therefore conclude that electrophysiological properties of hGFAP-EGFP+ cells in the ON (1) differ significantly from typical passive astrocytes in the forebrain and (2) comprise two electrophysiologically different populations.

### 2.2 Type 1 but not type 2 cells in the optic nerve express A-type potassium currents

The membrane properties seen in hGFAP-EGFP+ cells of optic nerve, particularly in type 1 cells, are reminiscent of ‘complex cells’ or NG2 glia. Such properties have been described in different brain regions of the hGFAP-EGFP transgenic mice before (Matthias et al., 2003; Graβ et al., 2004; Schools et al., 2003). The typical, slightly inactivating outward component consists, among others, of A-type potassium currents (Sontheimer et al., 1989, 1993; Steinhäuser et al., 1994). We used an established protocol (Sontheimer et al., 1989) to isolate A-type K^+^ currents of the hGFAP-EGFP+ cells in ON and forebrain (Fig. 1E and F). Currents were recorded in ON type 1 and type 2 cells (N = 21 mice), and in hGFAP-EGFP+ cells in cortex (N = 5 mice), by depolarizing voltage steps ranging from −60 mV to +20 mV starting from two different holding potentials, namely −70 mV (Fig. 1E, left) and −110 mV (Fig. 1E, middle). Subtraction of the currents starting at −70 from those at −110 mV resulted in currents that were characterized by rapid inactivation (Fig. 1E, right). Such A-type currents were found in the majority of ON type 1 cells (85%, n = 40) but were never detected in ON type 2 cells (n = 13; Fig. 1E and F), nor in cortical (n = 15; Fig. 1F) or hippocampal astrocytes (N = 4 animals, n = 9 cells; Suppl. Fig. 2A and B). The decay of the A-type currents had an average inactivation time constant of 20.59 ms (16.7/24.6 ms), and was comparable to those reported for cells considered at that time as oligodendrocyte precursors (Sontheimer et al., 1989) or complex cells (Steinhäuser et al., 1994). A-type currents in ON type 1 cells were sensitive to the K^+^ channel blocker 4-aminopyridine (4-AP). Application of 1 mM 4-AP caused a reduction of the current by 69.6% (N = 7 animals, n = 11 cells; Suppl. Fig. 2C and D).

### 2.3 NG2+ cells in optic nerve resemble electrophysiological properties of hGFAP-EGFP + type 1 cells

Given the presence of multiple NG2 cell-specific electrophysiological patterns in hGFAP-EGFP+ cells in optic nerve, we next used the NG2-YFP transgenic mouse model (Karram et al., 2008) to compare the electrophysiological properties of cells expressing NG2-EYFP in ON and cortex. We applied identical patch clamp protocols in optic nerves and cortex of age- and sex-matched NG2-EYFP mice (Fig. 2A). In ON, all NG2-YFP+ cells (N = 9 mice, n = 18 cells) had homogeneous membrane properties, thus, forming only one population (Fig. 2C and D). They closely resembled those of ON type 1 cells in hGFAP-EGFP mice in terms of outwardly rectifying I-V relationships (G_out_ = 7.3 (4.4/8.6) nS; G_in_ = 2.5 (0.2/6.9) nS, *p* = 0.0096) reversal potentials of −61.7 (−67.0/--53.3) mV (*p* = 0.078 vs. ON type 1 in hGFAP-EGFP + mouse) and membrane resistances of 177.2 (128.4/363.4) MΩ (p = 0.7354 vs. ON type 1 in hGFAP-EGFP + mouse). Furthermore, as for ON type 1 cells described above, A-type currents could be recorded in the majority of NG2+ cells in ON (76.9%, N = 9 mice; n = 13 cells; Fig. 2E and F), with an inactivation time constant of 20.89 ms (17.54/23.73 ms).

**Fig. 2:**
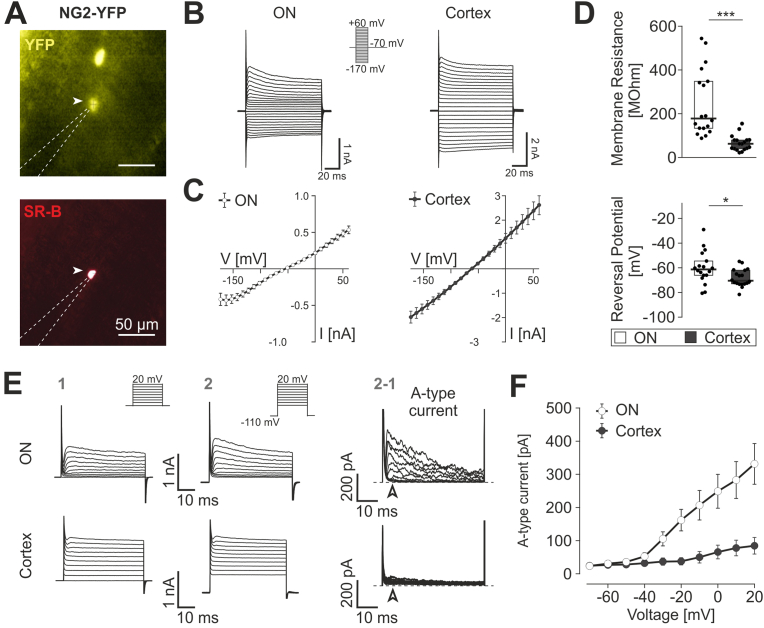
NG2+ cells in optic nerve resemble electrophysiological properties of hGFAP-EGFP+ type 1 cells **(A)** Fluorescent images of a patch-clamped cell (arrowhead) in an optic nerve longitudinal slice of a NG2-YFP mouse. *Top:* The transgenic YFP signal visible under fluorescent light. *Bottom:* same cell labelled with sulforhodamine B after 20 min of dialysis. Scale Bar: 50 μm. Dotted line: patch pipette. **(B)** Representative membrane currents of NG2-YFP+ cells during voltage pulses (*inset*) ranging from −160 mV to +60 mV in optic nerve and cortex. **(C)** Average current-voltage relationships of optic nerve (n = 18, left) and cortical NG2 glia (n = 18; right). **(D)** Box plots (median with 25/75% CI) of membrane resistances (upper panel) and reversal potentials (lower panel) for NG2-YFP+ cells in optic nerve (ON) and cortex. **(E)** Detection of A-type currents. Currents resulting from a voltage protocol as indicated by insets were recorded from optic nerve and cortical NG2-YFP+ cells (ON) from a holding potential of −70 mV (1) and a holding potential of −110 mV (2). The rapidly decaying A-type currents resulting from subtraction (2–1) of the currents collected from −70 mV from those collected from −110 mV was evident in ON but these currents were small in cortical NG2 glia. **(F)** Average current density of A-type currents in optic nerve (ON) (white, n = 13) and cortex (black, n = 15) plotted as a function of voltage.

Interestingly, basic membrane properties of NG2-YFP+ cells in the cortex (N = 9 mice, n = 18 cells) were different from the NG2-YFP+ cells in ON. Cortical NG2-YFP+ cells had outward and inward conductances of 18.5 (10.5/23.2) nS and 12.9 (10.5/23.2) nS, respectively. The median membrane resistance of cortical NG2-YFP+ cells was 60.5 (37.5/78.0) MΩ, and thus significantly smaller than in the ON counterparts (p < 0.0001 vs. ON NG2-YFP+), whereas the reversal potential was slightly more negative in cortex compared to ON (−70.9 (−73.6/-62.3) mV; *p* = 0.0300 vs. vs. ON NG2-YFP+; Fig. 2D). Furthermore, we did not observe A-type currents in the cortical NG2-YFP+ cells (Fig. 2F).

In summary, these data indicate that NG2_ON cells share electrophysiological properties with GFAP-EGFP+, ON type 1 cells.

### 2.4 The hGFAP-EGFP transgene is targeted to NG2-like cells in optic nerve

To test whether hGFAP-EGFP+ cells in optic nerve that were subject of our patch clamp approach, expressed the proteoglycan NG2, we performed immunohistochemical labelling in ON cryosections (Fig. 3A and B). Cells expressing the transgene EGFP were located preferentially close to the pia limitans, they extend processes in all directions, with most of them oriented in parallel to the longitudinal axis of the axons (Fig. 3A). They exhibited heterogeneous morphologies, as described earlier by Butt and colleagues (Butt et al., 1989, 1993). Notably, a large population of EGFP-expressing cells were also labelled for NG2 (Fig. 3B). Quantification revealed that 74 % of all EGFP-positive cells were also positive for the NG2 (n = 83 cells in 9 sections from N = 3 animals). Interestingly, this percentage of overlap matches well the percentage of ON type 1 cells (75%) with NG2-like membrane properties (see above). These findings were backed up by post-hoc labelling of patch-clamped and dye-filled cells of optic nerve with NG2 antibodies (Suppl. Fig. 3). Twelve out of n = 16 EGFP/biocytin positive cells that had been characterized as ON type 1 cells showed NG2 antibody labelling (75%; Suppl. Fig. 3B) whereas none of type 2 cells (n = 8) that lacked NG2-like electrophysiological features did label for NG2 after post-hoc staining ((N = 7 animals, Suppl. Fig. 3C and D). NG2-negative cells showed no apparent morphological difference compared to their NG2-expressing counterparts. In cortex, two morphological phenotypes of hGFAP-EGFP+ cells could be distinguished: cells with numerous, highly branched processes (like protoplasmic astrocytes) and more simply shaped cells that show either bi-or tripolar morphology as described previously (Nolte et al., 2001; Schipke et al., 2001). In contrast to ON, cortical hGFAP-EGFP+ cells did not exert NG2 co-expression (0%, 0 out of 73 GFAP-EGFP positive cells; Suppl. Fig. 4A and B).

**Fig. 3:**
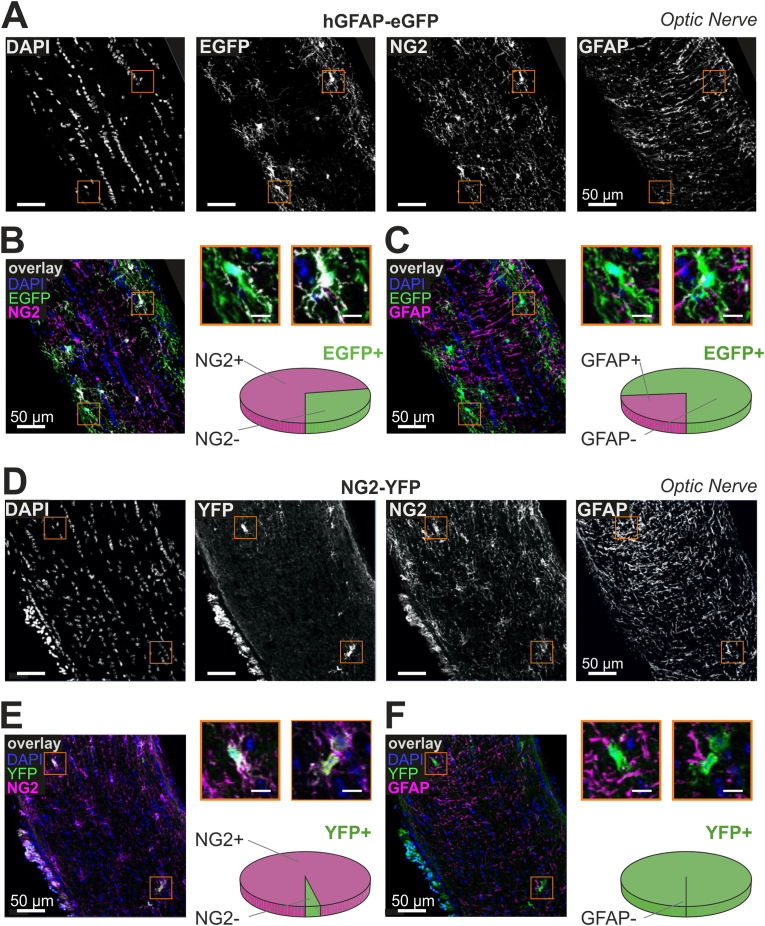
The hGFAP-EGFP transgene is targeted to NG2-like cells in optic nerve **(A)** Confocal images of a longitudinal optic nerve cryosection from a hGFAP-EGFP transgenic mouse. DAPI staining (left) shows typical “pearl cord” arrangement of glial cell nuclei. EGFP-positive cells (middle left) are often found close to the pia limitans. Processes extend preferentially in parallel to the axons. NG2 antibody (middle right) labels cell bodies and processes throughout the nerve. GFAP-labelled processes (right) typically stretched perpendicular to the longitudinal ON axis. Orange boxes refer to the magnified cells shown in panel B and C. Scale bars: 50 μm. **(B)** Overlay image of EGFP signal (green), NG2 (magenta) and DAPI (blue); same view field as in panel A. Overlapping signals appear in white. Smaller inset images (right top) are magnified views of two different EGFP + cells. Right bottom: The pie chart shows the percentage of EGFP+ cells that were positive (74%; magenta) and negative (26%; green) for NG2. Scale bars: 50 μm (large image), 10 μm (small images). **(C)** Same image as in (B) but overlaying EGFP (green), GFAP (magenta) and DAPI (blue). Note the sparse co-labelling of hGFAP-EGFP signals with endogenous GFAP. **(D**–**F)** Same representation as (in A-C) of the ON of a NG2-YFP transgenic mouse. Note that transgenic YFP signals strongly coincide with NG2 staining (E) but not with GFAP (F) (See also Figure S3, S4, S5). (For interpretation of the references to colour in this figure legend, the reader is referred to the Web version of this article.)

We also analysed if (endogenous) GFAP is colocalized with transgenic hGFAP-EGFP+ cells and performed immunolabeling with GFAP antibody in optic nerve and cortex (Fig. 3, Suppl. Fig.4). In optic nerve, GFAP immuno-labelling revealed a dense network of processes that appear knotted and irregular in shape as shown previously (Meyer et al., 2018). GFAP is mainly localized in processes which are oriented preferentially perpendicular to the axon direction whereas transgenic EGFP signal labels cytosol of individual cells and processes that stretch preferentially parallel to the axons’ direction (Fig. 3C, Suppl. Fig. 5). Quantification of overlap by the procedure described in Materials and Methods (Logiacco et al., 2021) revealed GFAP-labeling in about 25% of the EGFP+ cells (Fig. 3C). A 3D analysis with IMARIS in a subset of optic nerve specimens revealed that the average percentage of GFAP-stained volume co-localizing with EGFP is low (1.3%; n = 8 image stacks from N = 3 optic nerves; Supp. Fig. 5, supplementary information). In orthogonal views of optic nerve confocal stacks, GFAP labelled structures are rarely seen in cell profiles showing EGFP expression (Suppl. Fig. 5B). This unexpectedly low amount of hGFAP-EGFP co-labelling with endogenous GFAP can be explained by the fact the subcellular localization of GFAP protein is different from EGFP or that transgene is mostly targeted to other than GFAP positive astrocytes. In slices of cortex, GFAP immunolabeling was almost undetectable in most cortical layers (Suppl. Fig. 4A,C) whereas clearly expressed in subpial layers and white matter which is in accordance with the literature (Messing et al., 2020). To verify if the transgene expression in NG2 cells in the GFAP-EGFP mouse is region-specific - or is also found in other white matter regions - we tested whether hGFAP-EGFP+ cells in corpus callosum (CC) expressed the proteoglycan NG2 and performed immunohistochemical labelling in CC cryosections (Suppl. Fig. 6. Cells expressing the transgene EGFP exhibited heterogeneous morphologies. Notably, a large population of EGFP-expressing cells were also labelled for NG2 (Suppl. Fig. 6B). Quantification revealed that 73 % of all EGFP-positive cells were also positive for NG2 (n = 73 cells in 14 sections from N = 3 animals). NG2-negative cells showed no apparent morphological differences compared to their NG2-expressing counterparts. We also analysed if (endogenous) GFAP is colocalized with transgenic hGFAP-EGFP+ cells and performed immunolabeling with GFAP antibody in CC (Suppl. Fig. 6. GFAP immuno-labelling revealed a network of processes that were irregularly distributed along the callosal fiber tract. GFAP is mainly localized in processes, and quantification of overlap by the procedure described in Materials and Methods (Logiacco et al., 2021) revealed GFAP-labeling in about 45% of the EGFP+ cells which is more than in ON but still significantly less than in the cortex. In summary, we conclude that the majority of hGFAP-EGFP+ cells in the tested white matter regions express also the proteoglycan NG2 whereas endogenous GFAP expression is rather low.

Co-labelling of ON cryosections from transgenic NG2-YFP mice with GFAP also revealed no overlap of GFAP antibody labelling in cells that show NG2–YFP transgenic label (Fig. 3D–F). On the other hand, NG2 antibody labelling could be detected on the vast majority of EYFP-positive NG2 cells (Fig. 3E). Cell counting yielded 93.5% overlap for optic nerve and 93.8% for cortex (Suppl. Fig. 4). Of note, the amount of NG2-EYFP expressing cells in optic nerves is much lower than the number of cells that are labelled by NG2 antibody in the same model (51% in optic nerve and 56% in cortex), and the fine processes revealed by NG2 antibody staining are not visible (Fig. 3D).

In summary, the majority of cells expressing the hGFAP-EGFP transgene in optic nerve are positive for NG2 proteoglycan whereas they rarely express the classical astrocyte marker GFAP, suggesting that in optic nerves of hGFAP-EGFP transgenic mice the transgene is targeted mainly to NG2-like cells.

### 2.5 Cx43-CFP+ cells in on share passive membrane properties with cortical astrocytes and express GFAP

To identify astrocytes in the optic nerve, we used the Cx43kiECFP mouse line in which cyan fluorescent protein (CFP) expression is driven by the connexin43 (Cx43) promotor, a gap junction protein that is described to be astrocyte-specific (Wallraff et al., 2004). In adult ON, the Cx43-driven transgenic signal was found in perinuclear cytoplasm and on processes that stretch both in longitudinal and perpendicular direction and on endfeet lining the blood vessels. Immunolabeling with NG2 antibodies revealed distinct structures that did only partly overlap with Cx43-CFP signals (27.3%; n = 15 slices from N = 9 animals; Fig. 4A and B). The Cx43-CFP transgenic signal, however, strongly overlapped with GFAP immunostaining in ON (95.1%, n = 15 slices from N = 9 animals Fig. 4C). In cortex, there was only little overlap of NG2 antibody labelling with Cx43-CFP transgenic signal (8.2%; N = 4 slices from N = 3 animals; Suppl. Fig. 7B).

**Fig. 4:**
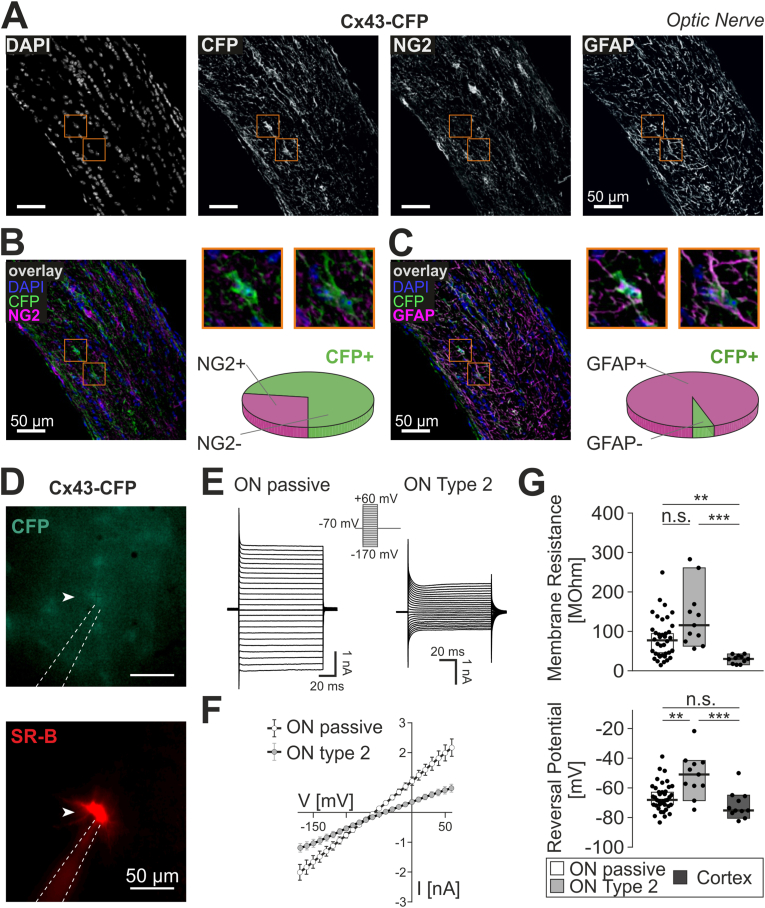
Cx43-CFP+ cells in ON share passive membrane properties with cortical astrocytes and express GFAP **(A)** Confocal images of a longitudinal optic nerve cryosection from a Cx43kiECFP transgenic mouse. DAPI staining (left) shows typical “pearl cord” arrangement of glial cell nuclei. CFP-positive cells (middle left) are found throughout the optic nerve, NG2 antibody (middle right) labels cell bodies and processes throughout the nerve. GFAP-labelled processes (right) typically stretch perpendicular to the longitudinal ON axis. Orange boxes refer to the magnified cells shown in panel B and C. Scale bars: 50 μm. **(B)** Overlay image of CFP signal (green), NG2 (magenta) and DAPI (blue), same view field as in panel A. Overlapping signals appear in white. Smaller inset images (right top) are magnified views of two different CFP + cells. The pie chart shows percentage of CFP+ cells that were positive (27.3 %; magenta) and negative (72.7% green) for NG2. Scale bars: 50 μm (large image), 10 μm (small images). **(C)** Same depiction as in (B) but overlaying CFP (green), GFAP (magenta) and DAPI (blue). Note the robust co-labelling of Cx43-CFP signals with endogenous GFAP (See also Figure S6). **(D)** Fluorescent images of a patched cell in the optic nerve of a Cx43kiECFP mouse. *Top:* The intrinsic Cx43-CFP signal visible under fluorescent light. *Bottom:* same cell labelled with sulforhodamine B after 20 min of dialysis. Arrows mark the cell; dotted line outlines the patch pipette. Scale bars: 50 μm. **(E)** Representative membrane currents of Cx43-CFP + cells during voltage pulses (*inset*) ranging from −160 mV to +60 mV in optic nerve (passive and type 2) (See also Figure S6). **(F)** Average current-voltage relationships of passive (n = 38, white) and type 2-like (n = 11; black) CFP+ cells. **(G)** Box plots (median with 25/75% CI) of membrane resistances (upper panel) and reversal potentials (lower panel) for Cx43-CFP+ cells in optic nerve (ON) and cortex. (For interpretation of the references to colour in this figure legend, the reader is referred to the Web version of this article.)

Patch-clamp analysis of the Cx43-CFP-positive cells in optic nerves (N = 19 animals, n = 49 cells) revealed that the majority of cells displayed passive current profiles, with no voltage-dependent components and linear IV curves (n = 38 out of 49 cells; 78%) (Fig. 4D–F), a median outward conductance of G_out_ = 13.1 (8.1/22.4) nS and a median inward conductance of G_in_ = 12.9 (7.9/24.8). In the remaining cell population (n = 11 out of 49 cells; 22%), we found slightly voltage-gated currents similar to those in hGFAP-EGFP + type 2 cells (G_out_ = 6.4 (4.8/11.2) nS, G_in_ = 8.4 (5.8/11.4) nS). Cx43-CFP+ cells with passive membrane properties in optic nerve did not differ significantly from their cortical counterparts. The latter represent a homogeneous population of passive cells with linear IV curves (N = 6 animals, n = 11 cells), with similar inward and outward conductances (G_out_ = 38.0 (26.9/47.0) nS; G_in_ = 25.6 (23.2/53.3) nS; Suppl. Fig. 7 5D,E). Both passive and more complex type-2 Cx43-CFP+ cells in ON displayed significantly higher membrane resistances as compared to cortical Cx43-CFP+ (passive cells ON: 79.6 (40.4/110.1) MΩ, complex cells ON: 119.1 (76.4/174.4) MΩ vs. cortex: 30.5 (17.4/39.4) MΩ; *p* = 0.0012 and *p* < 0.0001, respectively; Fig. 4G). Median reversal potential in optic nerve was −68.3 (−72.1/-60.2) mV for passive cells, which was significantly more negative than in type 2 optic nerve cells (−50.9 (−58.7/-42.8) mV; *p* = 0.0095). The reversal potential of membrane currents of more complex cells also differs significantly from median reversal potential of cells in cortex which is −75.5 (−77.3/-65.7) mV (*p* = 0.0005). Comparison of the reversal potentials of passive cells in ON and cortex revealed no difference. We also tested for the presence of A-type currents in optic nerve and cortical Cx43-CFP+ cells as described above and could not detect such current components in any of these cells (ON: N = 19 animals; ON passive: n = 27, ON type 2: n = 5; Cortex: N = 6 animals, n = 11 cells; Suppl. Fig. 8). Taken together, our immunohistochemical and electrophysiological data confirm the identity of optic nerve Cx43-CFP-positive cells as astrocytes.

### 2.6 Glial cells in the optic nerve are weakly coupled

To determine the efficiency of gap junction coupling of glial cells in ONs we dialyzed cells by whole cell patch clamp for 20 min with the gap junction-permeable tracer biocytin, as described previously (Maglione et al., 2010). Slices were subsequently fixed and stained with Cy3-streptavidin to identify biocytin spread. In the hGFAP-EGFP transgenic model, we analysed 68 ON slices from 31 animals. The majority of ON type 1 cells (37 out of 68 = 54%) showed no evidence of coupling and only the injected cell was labelled with biocytin (Fig. 5A). Similar results were obtained for type 2 cells; 9 out of 15 (60%) were not dye-coupled. In the remainder cells that were coupled, we found a median number of biocytin + cells of 2.0 (2.0/2.75) cells for ON type 1 (*n* = 16), and 4.0 (2.8/8.5) cells for ON type 2 cells (*n* = 6) (Fig. 5D). For ON type 1 cells, the tracer spread preferentially in the direction parallel to the axons (47.9 (11.9/67.1) μm) as compared to perpendicular direction (20.1 (10.8/32.4) μm, Wilcoxon test, *p* = 0.0210). In type 2 cells, the tracer spread 66.1 (33.7/118.3) μm along the nerve fibers and 14.8 (10/92.7) μm in the perpendicular direction, however the difference was below significance (Fig. 5C).

Accordingly, the surface area of the spread dye was also small and similar for type 1 and type 2 cells (resp. 153 (113/242) μm^2^, and 341 (201/947) μm^2^; Fig. 5B). Assessment of dye-coupling of GFAP-EGFP+ cells in cortex (N = 7 animals, *n* = 19 slices) by the same protocol resulted in much larger, mostly symmetric shaped networks with a median number of 87 (48/151) biocytin+ cells (Fig. 5C and D), a median surface area of the dye spread of 87612 (39365/92667) μm^2^ (Fig. 5B). In hippocampus, we found also large networks with a median of 96 (67/133) biocytin+ cells (N = 6 animals, n = 21 slices) and surface area of the spread of 30794 (24773/47066) μm^2^) after dye filling of GFAP-EGFP cells (Fig. 5B–D) confirming previous studies (Wallraff et al., 2004). Networks in ON were thus significantly smaller compared to cortex and in hippocampus in terms of number of incorporated cells (type 1 vs cortex: *p <* 0.0001, type 1 vs. hippocampus: *p <* 0.0001, type 2 vs. cortex: *p <* 0.0001, type 2 vs. hippocampus: *p <* 0.0001), as well as in terms of surface area of the dye spread (type 1 vs cortex: *p <* 0.0001, type 1 vs. hippocampus: *p =* 0.013, type 2 vs. cortex: *p* < 0.005).

**Fig. 5:**
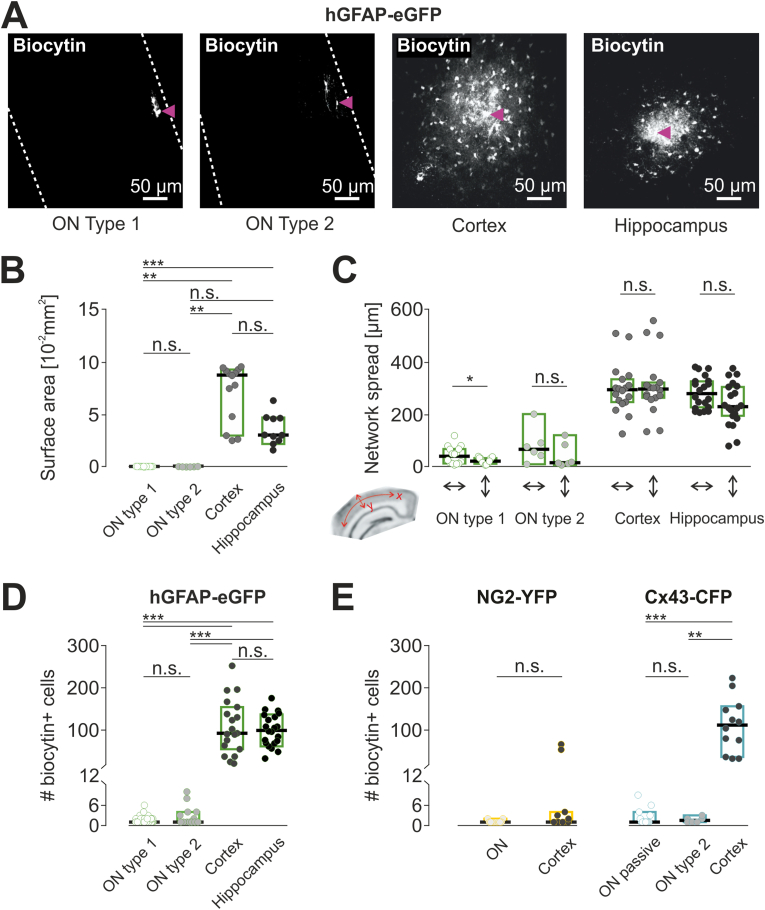
Glial cells in ON are weakly coupled **(A)** Examples of biocytin-filled networks after patch-clamp/dye-filling of hGFAP-EGFP expressing cells in ON (type 1 and type 2 cell), cortex and hippocampus. Dashed lines illustrate edge of the nerve. Scale bars 50 μm. **(B**–**D)** Analysis of coupled networks in ON (type 1: n = 37, type 2: n = 15), cortex (n = 19) and hippocampus (n = 21) of hGFAP-EGFP mice. Scatter plots depict median values of number of biocytin+ cells (B) surface area of biocytin+ cells (C) dye-spread in horizontal and vertical directions (D) median values of number of biocytin+ cells in hGFAP-EGFP mice **(E)** median values of number of biocytin+ cells in NG2-YFP mice (ON: n = 11, cortex: n = 12) and biocytin+ cells in Cx43-CFP mice (passive: n = 16, type 2: n = 6, cortex: n = 12) in optic nerve and cortical slices.

To characterize the cellular identity of the few ON cells coupled to the injected ones, we applied cell type-specific markers after patch clamp and tracer-filling of the cells in the GFAP-EGFP mouse model. We were able to detect Olig2, a transcription factor expressed by cells of the oligodendrocyte lineage (Zhou et al., 2000) in some of the networks in ON, both those originating from injection of a type 1 and a type 2 cell. Other biocytin+ cells were positive for the EGFP-GFAP transgene. The majority of biocytin+ cells, however, were unlabelled (data not shown). Interestingly, in another set of posthoc immunostaining experiments, NG2 antibody was applied to probe for NG2 immunolabeling in biocytin-filled cells, and to test if we can link the electrophysiological properties of ON type 1 and type 2 cells to the presence or absence of NG2: in 16 cells (N = 5 animals) that were characterized as ON type 1 cells two thirds showed NG2 immunolabeling. On type 2 cells (N = 5 animals, n = 8 cells), in contrast, we never detected NG2 immunolabeling (Suppl. Fig. 3).

While the nature of unlabelled cells could not be defined, these data indicate that in this white matter tract GFAP-EGFP+ cells form small networks with cells having both oligodendrocyte (Olig2), and NG2-like properties.

To determine the coupling in ONs of NG2-YFP mice, we performed tracer-fillings of NG2-YFP+ cells as described above. We analysed n = 11 slices of ON and n = 12 from cortex obtained from NG2-YFP animals (N = 9). In ON, we found coupling in only two slices, in both cases only two biocytin+ cells were found (Fig. 5E). In cortex, coupling was detected in 5 slices, the median network size considering only these slices was 4.0 (2.5/53.5), whereas median number of labelled cells including all 12 slices was only 1.0 (1.0/3.8) (Fig. 5E). This is not different from the number of labelled cells in ON.

In the Cx43kiECFP mice (N = 12) we performed biocytin-filling in 22 Cx43-CFP+ cells in ON and 12 cells in cortex. From 16 Cx43-CFP+ cells in ON with passive membrane properties, five (37.5%) were coupled, with a median biocytin+ network size of 4.0 (2.5/7.5) cells (Fig. 5E). From six cells expressing low-amplitude voltage-gated currents, coupling was observed in three slices (50%) and the median was 2 (2/3) biocytin+ cells. In contrast, all (100%) tracer-filled Cx43-CFP+ cells in cortex (n = 12) formed large coupled networks with a median network size of 107 (39/151) biocytin+ cells. Compared to the cortex, coupling was significantly lower in ON, both filling passive cells and cells with low-amplitude voltage gated currents (*p* < 0.0001, and *p* = 0.002, respectively) (Fig. 5E).

In ON slices, the median tracer spread after biocytin-filling Cx43-CFP+ cells was 38.0 (26.7/69.7) μm in parallel to the axons and 24.2 (13.6/45.3) μm in perpendicular direction for the passive cells (n = 5), and 41 (37.5/46.5) μm and 15.1 (10.7/15.4) μm, respectively, for the type 2 cells (n = 3). No analysis of asymmetrical spread was performed due to low n. As in the hGFAP-EGFP mice, biocytin labelled networks in cortex were roundish, and the tracer spread 46411 (22873/98076) μm in diameter.

In summary, in the ON slices we revealed very low glial cell coupling, irrespective whether we analysed cells with typical astrocyte characteristics (Cx43-CFP+, passive membrane currents), NG2-cells (NG2-YFP+; time- and voltage-dependent currents), or type 1 and type 2 ON cells in the GFAP-EGFP-model. At the same time, our control studies in cortical grey matter revealed considerably larger networks as previously described for astrocytes, with exception of NG2 glia in the NG2-YFP transgenic mouse, which are weakly coupled in cortex, if at all.

## 3 Discussion

In the current study we characterized the electrical properties and coupling of glial cells in the adult mouse ON. To assess the cells with the patch-clamp technique, we developed a novel slice preparation of ON. This preparation allowed for the first time the application of the whole cell patch clamp technique in this white matter region, and we were able to record membrane currents and dialyze the cell to identify a tracer spread to detect coupling. The ON is frequently used as a model tissue to study axon-glia interactions in the context of myelination, and to unravel the role of glia for axonal metabolism (Bolton et al., 2005; Nave et al., 2014; Morrison et al., 2013). Previous studies have shown the importance of astrocytic glycogen for maintenance of axonal activity (Saab et al., 2013). In another white matter region, the corpus callosum, we have previously shown that oligodendrocytes provide metabolic support for axons (Meyer et al., 2018) and that gap junction-coupled glial networks are crucial in this process. In the thalamus, astrocytes and oligodendrocytes jointly provide metabolites to maintain synaptic activity (Philippot et al., 2021).

Glial cells in the mammalian ON have been studied in nerve whole mounts and with sharp electrodes (Butt et al., 1989, 1993; Marrero et al., 1989; Brown et al., 2001). In such preparations, however, individual glial cells were not accessible for patch clamp studies due to the connective tissue/meninges surrounding the axonal tract. The longitudinal slice preparation presented here exposes the axons and makes the associated glia accessible for patch clamp recording and dye-filling. We used the hGFAP-EGFP transgenic mouse (Nolte et al., 2001) which has been widely used to identify astrocytes in different CNS regions. Previous studies in hippocampus and cortex showed that the majority of EGFP-positive cells is characterized by passive membrane properties, and intense coupling, typical for the classical astrocytes. However, these studies also identified a subpopulation of EGFP-positive cells with distinct electrophysiological, morphological and molecular features, obviously representing NG2 glia (Matthias et al., 2003; Graβ et al., 2004; Wallraff et al., 2004).

In ONs, we identified two subtypes of EGFP-fluorescent cells by patch clamp analysis; the more common one, type 1, had membrane properties similar as NG2-like cells from other CNS areas (Trotter et al., 2010; Steinhäuser et al., 1992; Lin et al., 2002) including inactivating, outwardly rectifying currents and the presence of A-type currents. These type 1 cells were positive for NG2 proteoglycan in posthoc immunostainings. The other subtype, type 2, lacked A-currents and did not label for NG2. In the NG2-YFP knock-in mouse model (Karram et al., 2008), the YFP-expressing cells displayed similar electrophysiological properties as the type 1 cells of the hGFAP-EGFP transgenic mouse model, i.e. high input resistance and A-type currents. In voltage clamp experiments in previous studies, NG2 glia, particularly in white matter, typically display such non-linear IV relationships characteristic of A-type and delayed rectifyer K^+^ channels (Sontheimer et al., 1989; Barres et al., 1990; Berger et al., 1991; Borges et al., 1995; Kettenmann et al., 1991; Williamson et al., 1997). The role of NG2 K^+^ channels mediating A-type currents has been assessed in detail in Sun et al. (2016) (Sun et al., 2016). In the hippocampus in situ, NG2 cells perform linear integration of glutamatergic synaptic input from neuronal cells through rapid Ca^2+^ signals mediated by low-voltage activated Ca^2+^ channels. Gating through these Ca^2+^ channels is under strict inhibitory control of NG2 A-type channels. Indeed, blocking A-type currents increased the amplitude as well as the threshold for Ca^2+^ responses through low-voltage activated Ca^2+^ channels. Prolonged synaptic input itself inactivates a fraction of A-type channels, thereby rendering a high frequency train of synaptic input that causes Ca^2+^ signaling in NG2 glia. These Ca^2^ signals, in turn, are a mechanism by which neurotransmitter release is coupled to activity-dependent myelination by NG2 cell differentiation. A-type K^+^ channels are thereby proposed to have important regulatory roles in how and to what extent neuronal activation affects oligodendrogenesis by NG2 cells (Sun et al., 2016). It is likely that in optic nerve, A-type channels on NG2 cells may serve a similar role, since it is well established that axons in the optic nerve release glutamate in an activity dependent-fashion (Saab et al., 2016) and that NG2 cells express glutamate receptors (unpublished results from our lab).

The electrophysiological similarity of the NG2-YFP+ and hGFAP-EGFP+ type 1 cells strongly suggests that in adult ON, the transgene of hGFAP-EGFP mice is targeted preferentially to NG2 glia rather than to GFAP-positive astrocytes, in contrast to cortex or hippocampus. Our data therefore shed doubt on the usability of common astrocyte reporter mouse strains in the ON. Similar findings were reported for the thalamus where glial cells also express unusual antigen profiles and form panglial coupled networks (Griemsmann et al., 2015), highlighting the importance of verifying transgenic lines across different studies and regions. This seems particularly true for the expression of EGFP under the human GFAP promotor, as other studies in hippocampus and the respiratory centers have demonstrated weak expression of EGFP in NG2 glia with complex current patterns (Matthias et al., 2003; Graβ et al., 2004). In these studies, however, strong EGFP expression was observed in passive cells, i.e. GFAP-positive and NG2-negative astrocytes. These studies have raised the question to which extent these are really distinct populations. Indeed, the transcription of GFAP-mRNA is active in both passive astrocytes and complex NG2-glia (Zhou et al., 2000), and many NG2 glia also express transcripts for S100b and GFAP as well as S100b protein (Moshrefi-Ravasdjani et al., 2017a). In our study, however, EGFP expression in ON was present in cells that were mostly negative for GFAP. Thus, in the same individual mouse, the hGFAP promotor seems to drive strong EGFP expression in astrocytes of hippocampus and cortex, but not ON. Indeed, we found that in another white matter region, the corpus callosum, the hGFAP promotor also targets NG2 cells, rather than GFAP-positive cells. This might reflect distinct intrinsic properties of the astrocytes in white matter as compared to grey matter regions. Different coupling and membrane currents further support this assumption. In the GFAP-EGFP mice, we also identified type 2 cells that were overlapping with a less common type in Cx43-CFP mice. These cells lacked A-type currents, but showed some voltage-gated current conductance. These cells were always NG2 negative, and may be a subtype of astrocytes that was in fact targeted in both transgenic mouse lines. The Cx43-CFP mice more reliably labelled cells in the ON that showed the classical electrophysiological properties of astrocytes, showing also a high overlap with endogenous GFAP. The majority of cells in the Cx43-CFP mice displayed passive linear current profiles, the electrophysiological properties typically associated with astrocytes. These properties are linked to one of the physiological functions of astrocytes, namely mediating K^+^ homeostasis requiring gap junction coupling and a high membrane permeability to K^+^ for spatial buffering of K^+^ (Wallraff et al., 2006).

Thus, in the ON, the distinction between NG2 glia and astrocytes largely fits the current framework that NG2 glia and astrocytes are distinct populations, based not only on the expression of immunocytochemical markers, but also on electrical properties. The passive-subtype cells may represent an intermediate subtype of astrocytes that are not clearly positive for GFAP, negative for NG2, and positive for Cx43, and with slight voltage dependence of their current profile. This fits the current consensus when pooling all studies assessing expression of NG2 and GFAP in ON. Overlap of these markers in the ON has been investigated in several studies before (Greenwood et al., 2003; Butt et al., 1999; Alghamdi et al., 2015; Bolton et al., 2006; Hamilton et al., 2009, 2010). Only one reported co-expression in double-immuno-labelled rat optic nerve sections and with immuno-gold labeling (Alghamdi et al., 2015).

We have furthermore found that the panglial coupling of ON astrocytes (as measured through Cx43-CFP and GFAP-EGFP type 2), as well as of ON NG2 glia (as measured through NG2-YFP and GFAP-EGFP type 1), is much lower as compared to grey matter regions, in our study as well as in other studies using biocytin dyalization to determine network size (Hösli et al., 2022, Pelz et al., 2024, Watanabe et al., 2023) (supplementary table 1). In astrocytes, >50% of the cells appeared uncoupled to any neighboring cell, whereas the biocytin labelled networks did never exceeded 8 cells. When we applied the same procedure to astrocytes in cortex or hippocampus as direct comparison, we observed much larger networks as previously described (Griemsmann et al., 2015). When compared to another white matter region, the corpus callosum, coupling in the ON was also considerably lower. While the panglial networks in corpus callosum were not as large as in the cortex, there was still significant coupling with an average network size of 13 biocytin+ cells when filled through astrocytes (Meyer et al., 2018) (supplementary table 1). In contrast, in spinal cord white matter the dye injected into an oligodendrocyte remained restricted to that cell (Pastor et al., 1998); astrocyte coupling was not evaluated. Using another technique, namely injection of dye with sharp electrodes, coupling was previously described for glial cells in the ON (Butt et al., 1989, 1993). However, the results of that method of dye-filling cannot be quantitatively compared to our study. It cannot be ruled out that the procedure of preparing slices may negatively affect the coupling rate. Moreover, the cells were not identified by cell-type specific markers at that time and relied on morphological identification and there was no direct comparison in parallel to grey matter preparations. The general difference of panglial coupling between grey and white matter may reside in distinct function of glial cells in these different tissues. In cortex, large astrocytic networks are instrumental for the delivery of metabolites to neurons to sustain activity (Rouach et al., 2008), whereas in the corpus callosum, metabolic support relies on coupled oligodendrocytes, rather than astrocytes (Meyer et al., 2018). In the thalamus, astrocytes and oligodendrocytes jointly supply metabolites through panglial coupling networks to neurons (Philippot et al., 2021). It is suggested that in ON, metabolic support of axons relies on oligodendrocytes, rather than astrocytes (Saab et al., 2016; Looser et al., 2024), and that this may not depend on panglial coupling to distribute energy substrates. Indeed, optic nerve axonal activity triggers glycolysis in oligodendrocytes through activation of Kir4.1 channels by increases in extracellular K+, and pharmacological or genetic inhibition of these channels causes metabolic deficits and axonopathy (Looser et al., 2024).

Similarly, whereas astrocytic coupling is critical for K+ clearance in grey matter, oligodendrocytes have also been shown to have K⁺ buffering abilities, and deletion of Kir4.1 channels on oligodendrocytes in white matter severely impairs K⁺ clearance and results in seizures (Larson et al., 2018). Similar to metabolic coupling, it appears that in the adult optic nerve, K⁺ homeostasis is not critically dependent on the glial syncytium. Instead, oligodendrocytes have been suggested to perform activity-induced K+ clearance in isolation (Hösli et al., 2022). These studies may partially explain the limited network sizes we found in optic nerve, particularly in comparison to grey matter.

NG2 glia were also coupled but much less than astrocytes and even oligodendrocytes. They were even uncoupled in the majority of experiments in ON and cortex. Limited coupling is obviously present which was seen both in the GFAP-EGFP type 1 cells, which we identified by NG2 immunolabeling within coupled networks, as well as in the NG2-YFP mouse. Coupling of NG2 glia is not a frequently reported phenomenon. Weak coupling of NG2 cells has been reported in corpus callosum (Maglione et al., 2010; Moshrefi-Ravasdjani et al., 2017b), but not in other regions (Griemsmann et al., 2015; Wallraff et al., 2004; Houades et al., 2008; Xu et al., 2014).

In conclusion, we found that glial cells in the ON show low coupling efficiency and require different tools for identification in physiologic experiments. Furthermore, the acute slice preparation developed and presented here allows for future single cell electrophysiological to assess glia cells in the optic nerve.

## Declaration of competing interest

The authors declare that they have no known competing financial interests or personal relationships that could have appeared to influence the work reported in this paper.

## Figures and Tables

**figs1:**
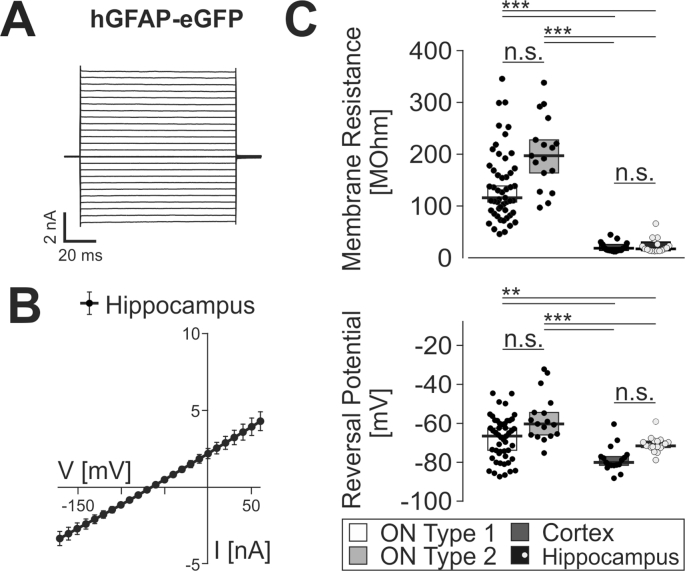


**figs2:**
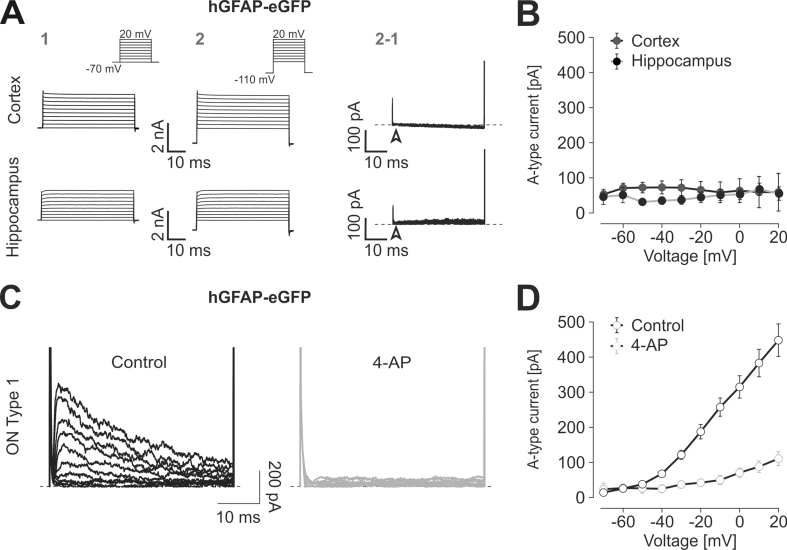


**figs3:**
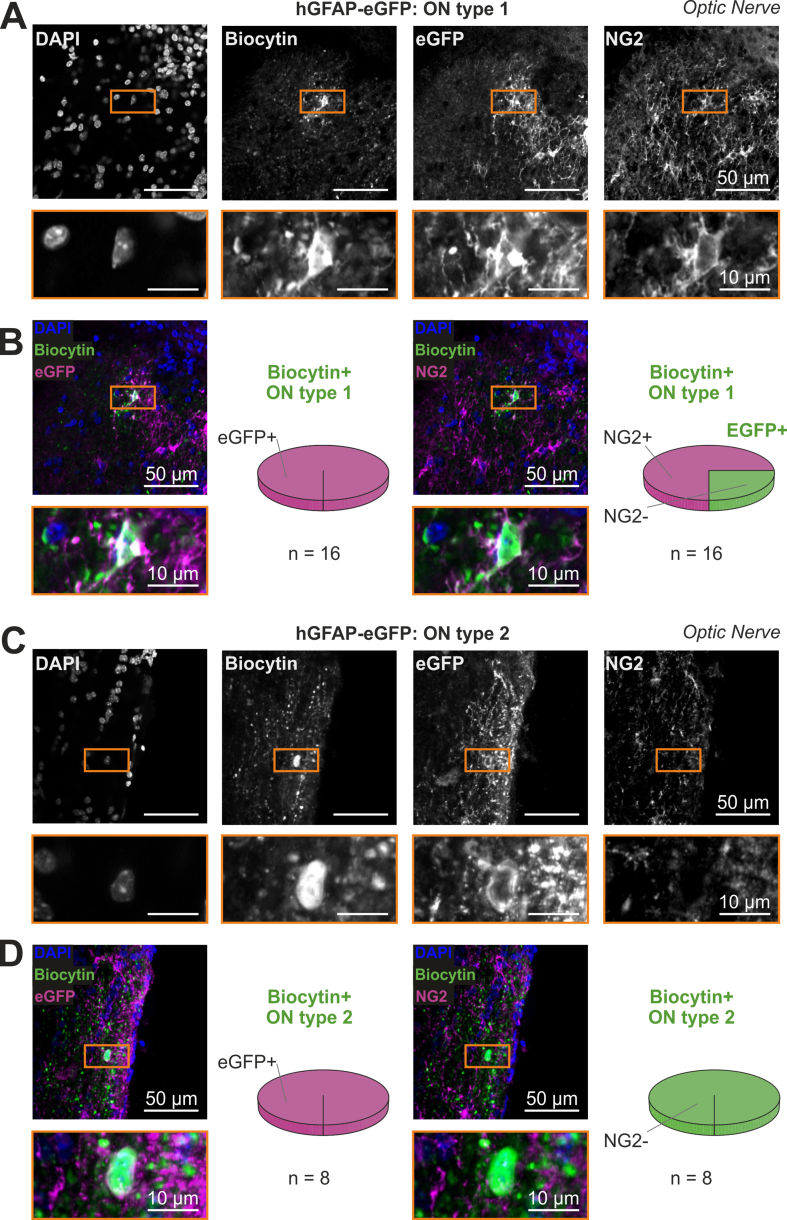


**figs4:**
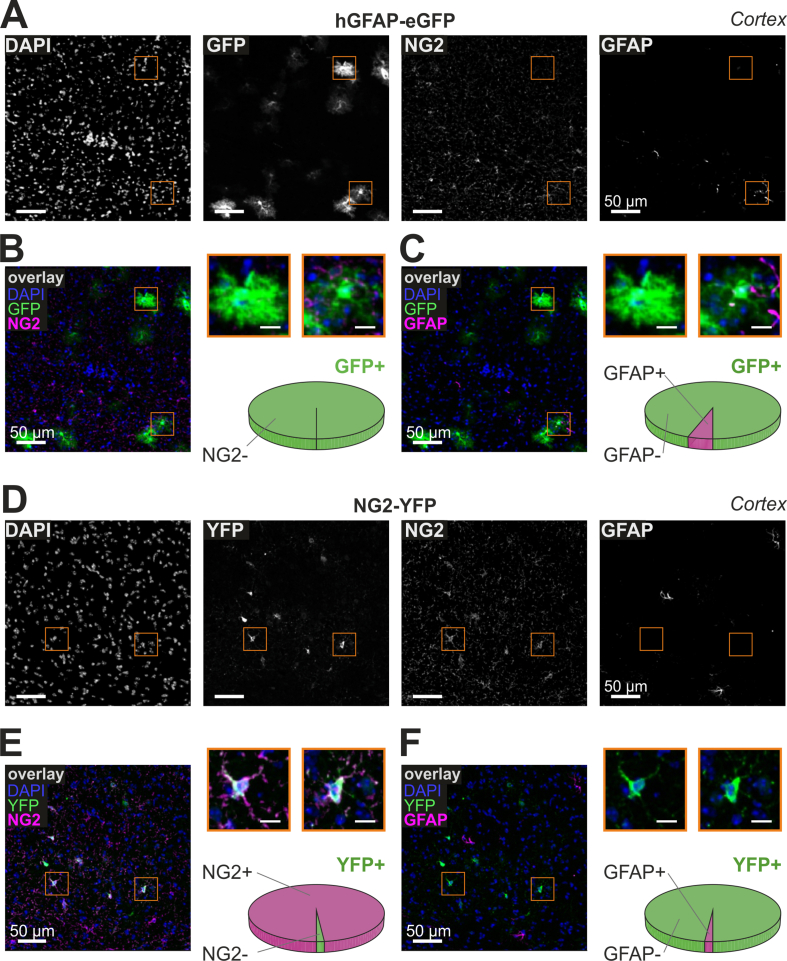


**figs5:**
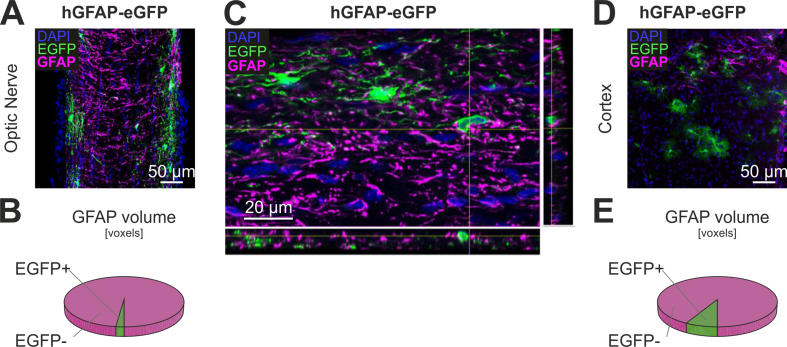


**figs6:**
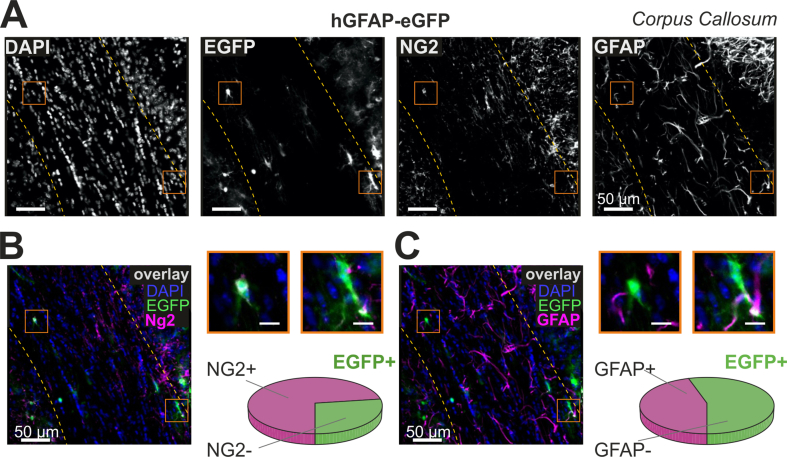


**figs7:**
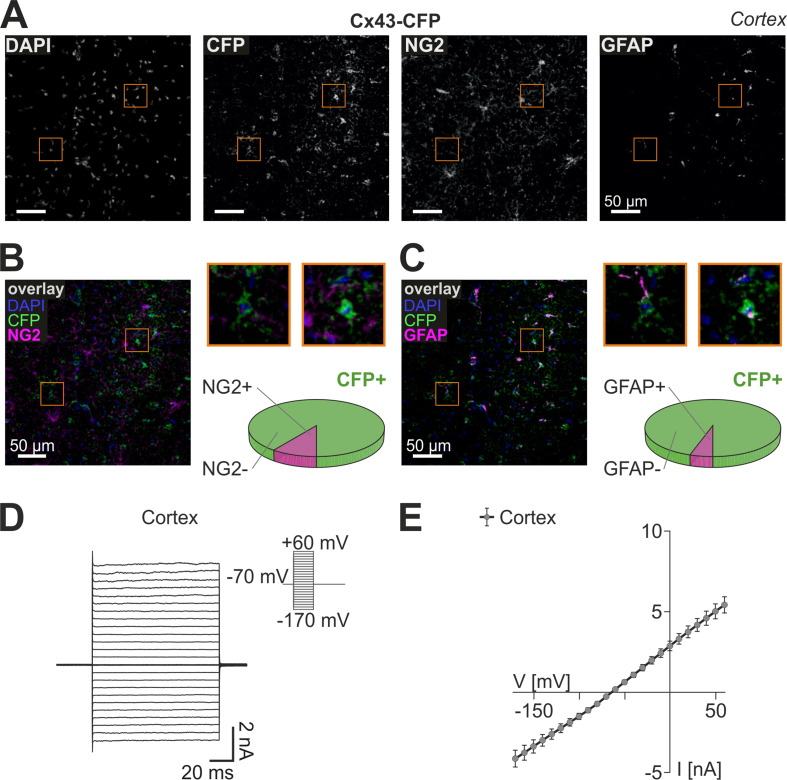


**figs8:**
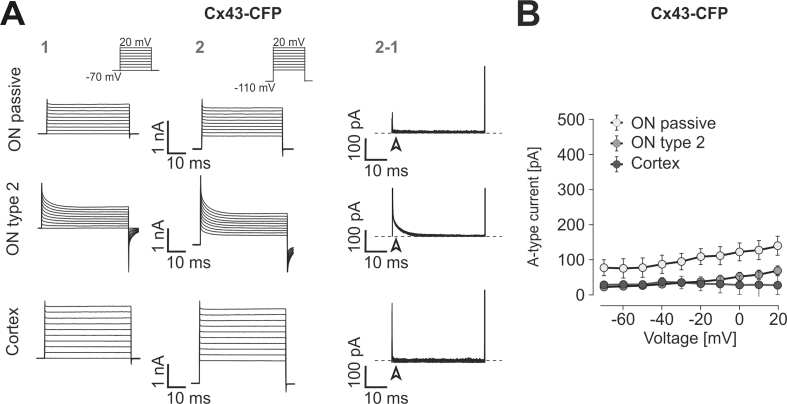


## Data Availability

Data will be made available on request.

## Methods

### Animals and handling

All mice were kept in the local animal facility of the MDC Berlin according to the German law for animal protection, under a 12h/12h dark-light cycle with food and water supplied ad libitum. All mouse strains had a C57BL/6 genetic background, were of both sexes and aged between 4 and 8 weeks. For studies on astrocytes, we used transgenic hGFAP-EGFP mice, expressing the enhanced green fluorescent protein EGFP in astrocytes under control of the promoter for human glial fibrillary acidic protein (GFAP) (Nolte et al., 2001), and the Cx43ki ECFP transgenic model in which one allele of gap junction protein connexin 43 (Gja1) was replaced by enhanced cyan fluorescent protein (ECFP) resulting in cell-type-specific expression of ECFP in astrocytes (Degen et al., 2012). For studies on NG2 cells we used the NG2-EYFP knock-in mouse (Karram et al., 2008) expressing yellow fluorescent protein (EYFP) under the NG2 promoter.

### Preparation of acute optic nerve, hippocampus and cortex slices

Acute frontal brain slices containing the cortex or hippocampus were prepared from 4 to 8 weeks old mice as described previously (Maglione et al., 2010). For preparation of acute optic nerve slices, we developed a novel slice preparation. Briefly, transgenic reporter mice were sacrificed by cervical dislocation and their optic nerves removed by pulling the eye from the socket using forceps and cutting off the nerve at the base under a dissection microscope (Olympus SZ40s, Hamburg, Germany) (Fig. 1). Nerves were then placed at the bottom of a Petri dish filled with 5% low-melting agarose (Bio&sell, Feucht, Germany, BS20.47.025) in Hank's balanced salt solution (Gibco, Thermo Fisher, Inc. Waltham, MA, USA, 14025092) supplemented with Ca^2+^ and Mg^2+^ at a temperature of 25° Celcius. After gelling, a square of agarose containing the optic nerve at the bottom was cut, and mounted in a chamber with ice-cold bicarbonate-buffered slicing solution composed of 230 mM sucrose, 26 mM NaHCO_3,_ 2.5 mM KCl, 1.25 mM NaH_2_PO_4_, 10 mM MgSO_4_, 0.5 mM CaCl_2_, and 10 mM glucose; pH 7.4. The solution was continuously gassed with carbogen (95% O_2_, 5% CO_2_). For the optic nerve, longitudinal slices of 150 μm were prepared at 4 °C using a vibratome (HM 650 V, Microm International GmbH, Walldorf, Germany). This effectively halves the nerves, or separated it in three sections, with the middle section being 150 μm and the outer sections being thinner, with unknown thickness. Slices were stored at room temperature (21 °C) for up to 5 h in artificial cerebrospinal fluid (ACSF) composed of 134 mM NaCl, 2.5 mM KCl, 1.3 mM MgCl_2_, 2 mM CaCl_2_, 1.26 K_2_HPO_4_, 26 mM NaHCO_3_ and 10 mM glucose; pH 7.4. ACSF was continuously gassed with carbogen. Brain slices were prepared and stored in the same solutions but sliced on the vibratome at a thickness of 250 μm.

### Patch-clamp recordings and dye loading

Brain and optic nerve slices were transferred to a chamber mounted on the stage of an upright microscope (Slicescope II, Scientifica Ltd., Uckfield, East Sussex, England) equipped with a 60× water immersion objective (Olympus, Hamburg, Germany). Slices were fixed using a U-shaped platinum grid. The chamber was continuously perfused with ACSF. The experimental patch-clamp set up was as described in (Richter et al., 2014; Wendt et al., 2017). GFAP-EGFP + cells were identified by their green fluorescence at excitation and emission wavelengths of 488 nm and 510 nm ± 10 nm, respectively. ECFP fluorescence in Cx43-kiECFP mice was observed with excitation and emission wavelengths of 433 nm and 475 ± 10 nm, and fluorescent cells in NG2-EYFP mice were visualized at excitation and emission wavelengths of 513 nm and 530 ± 10 nm, respectively.

For recording and for dye loading, patch pipettes with 4–8 MΩ resistance were pulled from borosilicate glass (1.5 mm outside diameter, 0.315 mm wall thickness) and filled with a solution containing 30 mM KCl, 10 mM Glucose, 1 mM MgCl_2_, 0.5 mM CaCl_2_, 100 mM potassium-gluconate, 10 mM Hepes, 5 mM EGTA, 0.5% biocytin and 3 mM Na_2_ATP; pH 7.3. Sulforhodamine B (10 μg/ml; Sigma-Aldrich, St. Louis, MI, USA) was added to the pipette solution, and intracellular access of the solution was confirmed by fluorescence illumination (586 ± 10 nm). Cells were passively dialyzed via the patch pipette for 20 min with the pipette solution supplemented with the tracer 0.5% N-biotinyl-l-lysine (Biocytin, Sigma-Aldrich). During passive dye-loading, membrane currents were determined by a series of de- and hyperpolarizing voltage pulses starting from a holding potential (HP) of −70 mV ranging from −160 to +50 mV (100 ms; 10 mV increments), using an EPC 10 patch-clamp amplifier and TIDA 5.25 software (HEKA Elektronik, TIDA (RRID:SCR_014582)) as described previously (Richter et al., 2014). We compensated online only for pipette capacity (C_fast_), whereas membrane capacity and series resistance (C_slow_) remained uncompensated. The calculated reversal potentials were corrected for the liquid junction potential (LJP) of the intracellular solution, which was −8.858 mV, determined by using Patcher's Power Tools (Patchers Power Tools (RRID:SCR_001950) and Igor Pro 7 software (IGOR Pro (RRID:SCR_000325). Membrane potentials in the IV-plots were not corrected for LJP. Only cells whose series resistance was not higher than 200 % at the end of the dialysis phase compared to the start were taken into account for electrophysiological and immunohistochemical analysis. After patch-clamp recording and dye loading the pipette was carefully removed from the cell in order to disrupt the patch. Slices were further processed as described below.

Detection of A-type currents was carried out through depolarizing the membrane from a HP of −70 mV to potentials ranging from −60 to +20 mV at 10 mV increments for 50 ms each, and subtracting the corresponding current responses from the currents activated by the same sequence of voltage steps, but from HP -110 mV, as described in (Sontheimer et al., 1989). Gating through K^+^ channels was tested by applying this protocol in a separate series of experiments before and 6 min after start of application of the K^+^ channel blocker 4-AP (5 mM, Abcam, Cambridge, UK). 4-AP was applied for 3 min.

### Dye coupling experiments, immunohistochemistry and quantification

After patch clamp and dye-loading, slices were fixed in a solution of 4% paraformaldehyde in 0.1 M phosphate buffered saline (pH 7.4) over-night at 4 °C. For visualization of biocytin filled networks and immunolabeling of glia specific antigens, slices were incubated in blocking solution containing 2% Triton X-100 (TX-100), 2% BSA and 8% normal donkey serum (NDS) in Tris-buffered saline (TBS) at pH 7.4 for 2.5 h at room temperature. Rabbit polyclonal anti-olig2 (1:700, Millipore Cat# AB9610, RRID:AB_570666) or rabbit polyclonal anti-NG2 (1:250, Millipore Cat# AB5320, RRID:AB_91789), and/or guinea pig polyclonal anti-GFAP (1:500, Synaptic Systems Cat# 173 004, RRID:AB_10641162), and chicken polyclonal anti-GFP (1:400, Abcam Cat# ab13970, RRID:AB_300798), to enhance the GFP, CFP or YFP signal) were diluted in TBS containing 1% TX-100; 2% BSA; 8% NDS. The floating slices were incubated with primary antibodies for 48 h at 4 °C. Primary antibodies and biocytin were washed off with TBS-T (TBS with 0.1 % BSA, 0.5% Tween) and visualized by application of streptavidin-Cy3 (1:200; Jackson ImmunoResearch Labs Cat# 016-160-084, RRID:AB_2337244), Alexa-Fluor 647 donkey anti-rabbit (1:200; RRID:AB_2492288) or Alexa-Fluor 647 donkey-anti guinea pig (1:200; RRID:AB_2340476) and Alexa Fluor 488 donkey anti-chicken (1:200; RRID:AB_2340375), for 2h at room temperature. 50 ng/ml DAPI (Sigma-Aldrich) was applied to label the nuclei. Secondary antibodies were purchased from Jackson ImmunoResearch, Hamburg, Germany. Slices were rinsed in TBS-T and mounted with Aqua Poly/Mount (Polysciences Inc., Washington, USA). As control the primary antibodies were omitted. No unspecific cross reaction of secondary antibodies was observed in control experiments.

To further characterize the glial cells in optic nerves, longitudinal, 16 μm thick cryosections were prepared on a cryostat and mounted onto slices (superfrostPlus, Thermo Fisher Scientific) as described previously (Nolte et al., 2001). Coronal sections of corpus callosum, cortex and hippocampus were prepared as controls.

Both the labelled slices and cryosections were scanned using the Zeiss LSM 700 confocal microscope (Zeiss, Germany) controlled by ZEN Digital Imaging software for Light Microscopy (RRID:SCR_013672). Z-stacks with 1 μm steps were acquired, using a 20x/0.8 Plan Apochromat (Zeiss, Oberkochen, Germany, 420650–9901) and a 40x/1.3 EC Plan Neofluar Oil Ph3 (Zeiss, 440451–9903) objective. Pinhole size was set to 1 Airy unit. Images were processed with the open source software Fiji, ImageJ-win64 (RRID:SCR_002285) using the cell counter plugin and z-axis projection functions. Biocytin spread along longitudinal (X) and perpendicular (Y) to the main axon paths were measured using the “straight line” tool and ROI manager.

### Quantification of immunohistochemical stainings

Overlap of signals in cells from ON, cortex and hippocampus of GFAP-EGFP animals was quantified from 8bit 3D images using a custom-built procedure in IGOR Pro 6.37 (WaveMetrics; RRID:SCR_000325) as previously described in (Logiacco et al., 2021). Briefly, a prismatic volume of interest was manually set over a perisomatic region of an EGFP + cell. Voxels were binarized by using appropriate thresholds, and *EGFP* -positive voxels used as a mask. A cell was counted as *GFAP* + or *NG2*+ when at least 10% of *EGFP* + voxels were positive for the respective protein signals. The reason why we choose that rather low percentage of overlapping voxels to define a positive cell was due to the fact that at least GFAP protein is localized at different subcellular sites as compared to EGFP (See Suppl. Fig. 5). Image analysis of staining from NG2-YFP and Cx43-CFP animals was performed in a similar fashion. Images as shown in the figures were processed and arranged with ImageJ (RRID:SCR_003070) and CorelDraw (RRID:SCR_014235).

### Data analysis

All electrophysiological recordings were processed by using an in-house written software in IGOR Pro 6.37 (WaveMetrics; RRID:SCR_000325) (Wendt et al., 2017). IV curves were calculated and the following properties of each cell at start and finish of the dialysis were determined: series resistance, membrane resistance, membrane capacitance and the reversal potential. To that end, membrane currents were averaged for quantification between 50 and 95 ms after clamping the membrane to a given value from the resting potential. Membrane capacitance was calculated based on an exponential fit of the current decay in a response to a 10 ms test pulse. The same pulse was used to quantify the series resistance from the peak amplitude of the membrane capacitance currents (Wendt et al., 2017). Inward (G_in_) and outward (G_ou_) conductance was calculated from the current slope between −170 and −110 mV and 0 and + 60 mV, respectively.

### Experimental design and statistical analysis

Igor Pro 6.37 (WaveMetrics; RRID:SCR_000325) and GraphPad Prism 7 (RRID:SCR_002798) were used for statistical analysis. Results in the text and graphs are reported as median with 25%/75% CI, unless otherwise stated; n = number of cells/brain slices; N = number of animals. Normal distribution for all data was tested through D'Agostino-Pearson testing. With most data not normally distributed, we analysed all data non-parametrically. Data comparing more than two independent variables was subsequently tested using Kruskal-Wallis test with Dunn's post-hoc corrections. Comparisons between two independent variables were tested with Mann-Whitney U tests. Statistical test for each experiment is described in the figure legends. Differences were regarded to be significant at p < 0.05 (*), p < 0.01 (**), p < 0.001 (***) or p < 0.0001 (****).
