# Supplementary material for: Membrane properties and coupling of macroglia in the optic nerve
Source: Curr Res Neurobiol. 2024 Aug 14;7:100137. doi: 10.1016/j.crneur.2024.100137 (PMC11382002; doi:10.1016/j.crneur.2024.100137)
Supplement: Multimedia component 1 [file mmc1.docx]

**Supplementary Information**

**Supplementary Methods**

The 3D Imaris interactive software package (version 9.9.0, Bitplane, Oxford Instruments, RRID:SCR_007370) equipped with the colocalization plugin was used to analyze for overlap of GFAP- immunolabeling in hGFAP-EGFP positive structures in Z-stacks or confocal sections of fluorescently labelled cryosections of optic nerves. ROIs/volumes with transgenically labelled structures were selected arbitrarily and “% of volume A (= GFAP-immunolabeling) above threshold colocalized with volume B (transgene)” was generated as an output value.

**Supplementary Tables**

| **Author, year** | **Astrocyte identification** | **Age** | **Region: average number of tracer-labelled cells** |
| --- | --- | --- | --- |
| Pelz et al., 2024 [75] | Sulforhodamine B | P56-P70 | Cortex: 101  Hippocampus: 118 |
| Hösli et al., 2022 [67] | GLAST-CreERT2 mouse | P56-P70 | Hippocampus: ~150 cells |
| Watanabe et al., 2022 [76] | Sulforhodamine B | P20-P30 | Cortex: ~60 cells |
| Meyer et al., 2018 [21] | hGFAP-mRFP mouse | P28–35 | Corpus callosum: 13 cells |
| Claus et al., 2018 [20] | GFAP-EGFP mouse | P14-P17 | Thalamus: 35 cells |
| Griemsmann, 2015 [15] | GFAP-EGFP mouse | P30-P60 | Thalamus: ~110 cells |
| Griemsmann, 2015 [15] | Cx43-ECFP mouse | P30-P60 | Hippocampus: 79 cells  Cortex: 75 cells  Thalamus: 79 cells |
| Wallraff et al., 2004 [39] | GFAP-EGFP mouse | P9-P30 | Hippocampus: 120 cells |
| Houades et al., 2008 [69] | Current profile | P20 | Cortex: 57 cells |

**Supplementary table 1:** summary of average number of tracer-labelled cells in patch clamp studies in white and grey matter regions using dialyzation of biocytin in astrocytes identified through transgenic labeling, or by typical current profile, or by labeling with sulforhodamine B

**Supplementary Figure Legends**

**S1: Current profiles, IV curves, and membrane properties of GFAP-EGFP cells in hippocampus**

**(A)** Representative membrane currents of a hippocampal hGFAP-EGFP+ cell during voltage pulses (inset) ranging from 160 mV to +60 mV.

**(B)** Average current-voltage relationships of hippocampal hGFAP-EGFP+ cells (*n*=18).

**(C)** Box plots (median with 25/75% CI) comparing the membrane resistances (upper) and reversal potentials (lower) for hGFAP-EGFP+ cells in optic nerve (type 1: n=51, type 2: n=17), cortex (*n*=17) and hippocampus (*n*=18).

**S2: Characterization of A-type currents in hGFAP-EGFP mice**

**(A)** Currents resulting from a voltage protocol as indicated by insets were recorded from hGFAP-EGFP+ cells in cortex and hippocampus from a holding potential of -70 mV (1) and a holding potential of -110 mV (2). No A-type currents were found after subtraction.

**(B)** Average currents in cortex (black, n=15) and hippocampus (n=9) plotted as a function of voltage after subtraction reveals no A-type current.

**(C**) Example traces recorded from in optic nerve type 1 cells in hGFAP-eGFP mice, indicate currents after subtraction at baseline in control (black) and after application of 4-AP (light grey).

(**D**) Average current density in optic nerve type 1 cells at baseline (black) and in the presence of 4-AP (grey) (*n*=11).

**S3: Post-hoc immunolabeling of patched hGFAP-EGFP positive cells in optic nerve reveals NG2 labeling on cells that had been characterized as ON type 1, but not on those with ON type 2 characteristics.**

(A) Confocal images of an acute optic nerve slice from a hGFAP-EGFP transgenic mouse after patch-clamp and dye-filling of a cell with ON type 1 membrane characteristics. DAPI, Biocytin, EGFP, and NG2 signals are shown in grayscale as indicated. Scale Bar: 50 µm. Magnified views of the patched cell (as marked by frames) are given below. Scale Bar: 10 µm.

(B) Overlay image of biocytin signal (green), EGFP (magenta) and DAPI (blue), same view field as in panel A. Overlapping signals appear in white. Magnified view of the biocytin-filled cell below to show overlap with EGFP. Pie chart shows that all biocytin-filled ON type 1 cells (n=16) were eGFP positive. Right: Same cell but overlaying biocytin (green), NG2 (magenta) and DAPI (blue). Pie chart shows percentage of all biocytin+ ON type 1 cells that were positive (magenta) and negative (green) for NG2.

(C) and (D) Same depiction as in (A) and (B) but posthoc-labeling and quantification of NG2-overlap of n=8 cells with ON type 2 membrane characteristics.

**S4: Distribution of GFAP and NG2 in cortex of hGFAP-EGFP and NG2-YFP transgenic mice**

(A) Confocal images of a cortical cryosection from a hGFAP-EGFP transgenic mouse. DAPI, EGFP, NG2 and GFAP signals are shown in grayscale as indicated. Orange Boxes refer to the magnified cells shown in panel B and C. Scale bars: 50 µm.

(B) Overlay image of EGFP signal (green), NG2 (magenta) and DAPI (blue), same viewfield as in panel A. Overlapping signals appear in white. Smaller inset images (right top) are magnified views of two different EGFP+ cells. Right bottom: Percentage of EGFP+ cells (n = 73) that were positive (magenta) and negative (green) for NG2. Scale bars: 50 µm (big image), 10 µm (small images).

(C) Same depiction as in (B) but overlaying EGFP (green, n = 73), GFAP (magenta) and DAPI (blue). Note the sparse endogenous GFAP signals.

(D-F) Same representation as (in A-C) but the cortex of a NG2-YFP transgenic mouse was analyzed (n = 57 cells).

**S5: Voxel-based analysis of GFAP- and EGFP-positive structures in optic nerves and cortex of hGFAP-EGFP transgenic mice reveals only sparse overlap**

**(A)** confocal section of ON longitudinal cryosection showing EGFP+ transgene (green) and GFAP immunolabeled structures (magenta). EGFP signal (green) shows labeling of individual cells and processes that stretch preferentially parallel to the axons direction, and GFAP labelled processes extend mainly perpendicularly.

**(B)** Quantitative result of Imaris colocatization showing the average percentage of GFAP volume (black) that colocalizes with transgenic signal (hGFAP-EGFP) in ON and cortex.

(**C**) section and orthogonal views of Z-stack from ON of hGFAP-EGFP transgenic mouse; GFAP labeled processes (magenta) show little overlap with transgenic signal. Nuclei are stained by DAPI (blue).

(**D –E**) same representation as A, B, but for cortex.

**S 6: Distribution of GFAP and NG2 in corpus callosum of hGFAP-EGFP mice**

(A) Confocal images of a cortical cryosection from a hGFAP-EGFP transgenic mouse. DAPI, EGFP, NG2 and GFAP signals are shown in grayscale as indicated. Orange boxes refer to the magnified cells shown in panel B and C. Scale bars: 50 µm.

(B) Overlay image of EGFP signal (green), NG2 (magenta) and DAPI (blue), same field of view as in panel A. Overlapping signals appear in white. Smaller inset images (right top) are magnified views of two different EGFP+ cells. Right bottom: Percentage of EGFP+ cells (n = 73) that were positive (magenta) and negative (green) for NG2. Scale bars: 50 µm (big image), 10 µm (small images).

(C) Same depiction as in (B) but overlaying EGFP (green, n = 73), GFAP (magenta) and DAPI (blue).

**S7: Cortical data on Cx43kiECFP transgenic mice**

**(A)** Confocal images of a cortical cryosection from a Cx43kiECFP transgenic mouse. DAPI, CFP, NG2 and GFAP signals are shown in grayscale as indicated. Orange Boxes refer to the magnified cells shown in panel B and C. Scale Bar: 50 µm

**(B)** Overlay image of CFP signal (green), NG2 (magenta) and DAPI (blue), same viewfield as in panel A. Overlapping signals appear in white. Smaller inset images (right top) are magnified views of two different CFP+ cells. Right bottom: Percentage of CFP+ cells that were positive (magenta) and negative (green) for NG2.

**(C)** Same depiction as in **(B)** but overlaying CFP (green), GFAP (magenta) and DAPI (blue). Note the sparse endogenous GFAP signals.

**(D)** Representative membrane currents of a cortical Cx43-CFP+ cell during voltage pulses (inset) ranging from 160 mV to +60 mV.

**(E)** Average current-voltage relationships of cortical Cx43-CFP+ cells (black, n = 11) .

**S8: Detection of A-type currents in Cx43kiECFP transgenic mice**

**(A)** Currents resulting from a voltage protocol as indicated by the insets were recorded from optic nerve and cortical Cx43-CFP+ cells (ON passive and ON type 2) from a holding potential of -70 mV (1, top) and a holding potential of -110 mV (2, middle). A-type currents resulting from subtraction (2-1, bottom) of the currents collected from -70 mV from those collected from -110 mV were not detected in Cx43-CFP+ cells.

**(B)** Average A-type currents in ON passive (black, n=27), ON type 2 (light grey, n=5) and cortex (dark grey, n=11) plotted as a function of voltage.
